# Characterizing the One Health workforce to promote interdisciplinary, multisectoral approaches in global health problem-solving

**DOI:** 10.1371/journal.pone.0285705

**Published:** 2023-05-16

**Authors:** Eri Togami, Casey Barton Behravesh, Tracey V. Dutcher, Gail R. Hansen, Lonnie J. King, Katharine M. Pelican, Jonna A. K. Mazet

**Affiliations:** 1 One Health Institute, University of California, Davis, Davis, CA, United States of America; 2 National Center for Emerging and Zoonotic Infectious Diseases, U.S. Centers for Disease Control and Prevention, Atlanta, GA, United States of America; 3 Animal and Plant Health Inspection Service, U.S. Department of Agriculture, Riverdale, MD, United States of America; 4 Hansen Consulting LLC, Washington, DC, United States of America; 5 College of Veterinary Medicine, Ohio State University, Columbus, OH, United States of America; 6 Department of Veterinary Population Medicine, University of Minnesota, St Paul, MN, United States of America; Ethicon Endo Surgery, UNITED STATES

## Abstract

**Background:**

In recognition of the interconnected nature of complex challenges such as COVID-19, a collaborative, multisectoral, and transdisciplinary approach, referred to as One Health, has been employed to address sustainable development and strengthen global health security. Although significant investments have been made to build global health capacity, characterization of the One Health is absent from the literature.

**Methods and findings:**

We collected and analyzed perspectives from students, graduates, workers, and employers in One Health through a multinational online survey across health disciplines and sectors. Respondents were recruited through professional networks. A total of 828 respondents from 66 countries participated, representing governmental and academic institutions and students, among others; 57% were female, and 56% had completed professional health degrees. Interpersonal communication, communication with non-scientific audiences, and the ability to work in transdisciplinary teams were valued in the workplace and were considered essential competencies to build an interdisciplinary health workforce. Employers indicated difficulty recruiting workers, while workers indicated limited availability of positions. Employers identified limited funding and ill-defined career pathways as prominent challenges for retaining One Health workers.

**Conclusions:**

Successful One Health workers use interpersonal skills and scientific knowledge to address complex health challenges. Aligning the definition of One Health will likely improve the matching of job seekers and employers. Encouraging the employment of the One Health approach for a diverse range of positions, even if they do not explicitly include “One Health” in the job title, and clarifying the expectations, roles and responsibilities within a transdisciplinary team will lead to building a stronger workforce. As One Health has evolved to address food insecurity, emerging diseases, and antimicrobial resistance, it holds promise for supporting an interdisciplinary global health workforce that can make substantial progress on Sustainable Development Goals and improve global health security for all.

## Introduction

The COVID-19 pandemic has shown how a single pathogen can impact the lives of people around the world, overwhelm health systems, and damage economies. In fact, emerging infectious diseases, loss of biodiversity, decreases in food crop production leading to food insecurity, and increased severity and frequency of extreme weather events and their related health challenges, including mental health challenges, are just some of the serious threats to sustainable development and global health security [1–4]. In recognition of the interdependence and interconnected nature of these complex challenges, collaborative efforts have begun to be employed to respond to population growth, land-use change, climate change, increased movement of people and animals, and other drivers of impaired global health and functionality [5, 6]. To address sustainable development and global health security, we must work together to employ the One Health approach, defined as “a collaborative, multisectoral, and transdisciplinary approach–working at the local, regional, national, and global levels–with the goal of achieving optimal health outcomes recognizing the interconnection between people, animals, plants, and their shared environment” [7]. COVID-19 intensified the world’s attention on One Health, which has been endorsed by key entities, including the World Bank, World Health Organization, United Nations Environment Programme, the Group of Seven political forum (G7), as a critical approach for preventing and controlling for future health threats [8–11].

This approach will be most usefully implemented by strengthening the interdisciplinary health workforce. The health workforce plays an essential role in detecting, preparing for, responding to, and recovering from natural or human-made hazards and risks, including those related to climate change, and is essential to the maintenance of resilient communities and systems [12]. The Sustainable Development Goals (SDGs) 2030 [2], which seek “peace and prosperity for people and the planet, now and into the future,” highlight 17 essential global goals to advance health equity and well-being for all people and call for experts in the global health community to work together across sectors to bridge these gaps. In addition, the Global Health Security Agenda (GHSA), launched by a consortium of countries in 2014, prioritizes workforce development to address global infectious disease threats. The GHSA emphasizes the importance of developing a strong interdisciplinary, multisectoral workforce, including livestock professionals, laboratory scientists, biostatisticians, veterinarians, field epidemiologists, and physicians who can cooperate to help countries meet core capacities in line with the International Health Regulations (2005) and Performance of Veterinary Services [13–16].

Significant investments have been made to build global health capacity. More than 80 countries have access to graduates of Field Epidemiology Training Programs (FETP) and have established a global workforce of interdisciplinary epidemiologists numbering upwards of 18,000 [17, 18]. Since 2009, the Emerging Pandemic Threats Program of the United States Agency for International Development has championed efforts to train and employ a One Health workforce aiming to strengthen disease surveillance, preparedness, and response activities in human, animal, plant, and environmental health sciences, with over 21,000 One Health professionals trained in over 30 countries and 15 national One Health platforms launched or reinvigorated [19–21]. These programs, complemented by efforts of universities to encourage a more holistic approach to global health problem solving, have dramatically increased the number of professionals working in fields relevant to One Health over the past two decades [22].

Despite the recognition of the importance of One Health by international organizations and governments, the One Health workforce remains uncharacterized. To the best of our knowledge, there is no information about who makes up the One Health workforce and what their lived experiences have been. Workforce characterization efforts have most often focused on a single sector or discipline, such as human medicine, public health, veterinary medicine, infectious disease prevention and control, or nursing and midwifery [23–27]. Because of its multidisciplinary nature, characterizing the One Health workforce is complex; One Health is not an occupation but an approach to complex health problem-solving. Therefore, there is no accreditation of training, such as in public health, and there are no centralized registries for One Health workers, such as is common for in dentistry or veterinary medicine. As the first step to fill this gap, we sought to collate perspectives from students, graduates, workers, and employers in One Health through a multinational survey across multiple disciplines and sectors relevant to health. The objectives of this formative study were to build foundational knowledge of the One Health workforce regarding demographics, education, and employment, as well as to explore the benefits of One Health education to the workforce, the unique challenges that One Health workers face, and whether employers are satisfied with their employees working in One Health.

## Methods

### Survey development, structure, and distribution

To remedy the dearth of information regarding the One Health workforce in the literature, a survey was designed to begin to characterize the demographics, academic training, useful skills and experiences of the One Health workforce. Prior to survey development, there were several rounds of discussion and review among a survey committee made up of seven experts affiliated with the One Health Action Collaborative of the United States National Academy of Sciences, Engineering, and Medicine (NASEM), including two government officials, four academics, and one public health practitioner, all of whom were doctors of veterinary medicine with additional masters (Master of Science in Epidemiology, Master of Public Health in Epidemiology, Master of Preventive Veterinary Medicine, and Master of Public Administration) and doctoral degrees (Doctor of Philosophy in Epidemiology, and Doctor of Public Health). The survey committee developed a cross-sectional, questionnaire-based, multinational survey which was disseminated online (see S1 File for the full questionnaire). Investigators developed and distributed the 53-question survey using the online survey platform, Qualtrics ® (Utah & Washington, USA) similar to other scholarly workforce surveys [28]. Questions were structured with binary choice, multiple-choice, or text entry responses. Given the absence of a centralized workforce registry or any workforce characterization data, participants were recruited by non-probability sampling. This sampling method was necessary because there was no other way to identify One Health workers. A standardized questionnaire instrument was used, asking precisely the same questions to all respondents in an identical format and recording all responses in a uniform manner to maximize reliability [29]. To make the survey as accessible and valid as possible across cultural contexts, we used wording that was most consistent across countries and educational systems. For example, we used “post-secondary education” and “professional degree” and provided their definitions, avoided US-centric wording such as “undergraduate”, “college”, “doctoral degree”, and included specific examples of degrees by their discipline, such as “Professional degree in medicine, veterinary medicine, dentistry, or pharmacology” and “Research-based doctoral degree (Doctor of Philosophy (PhD))”).

**Fig 1:**
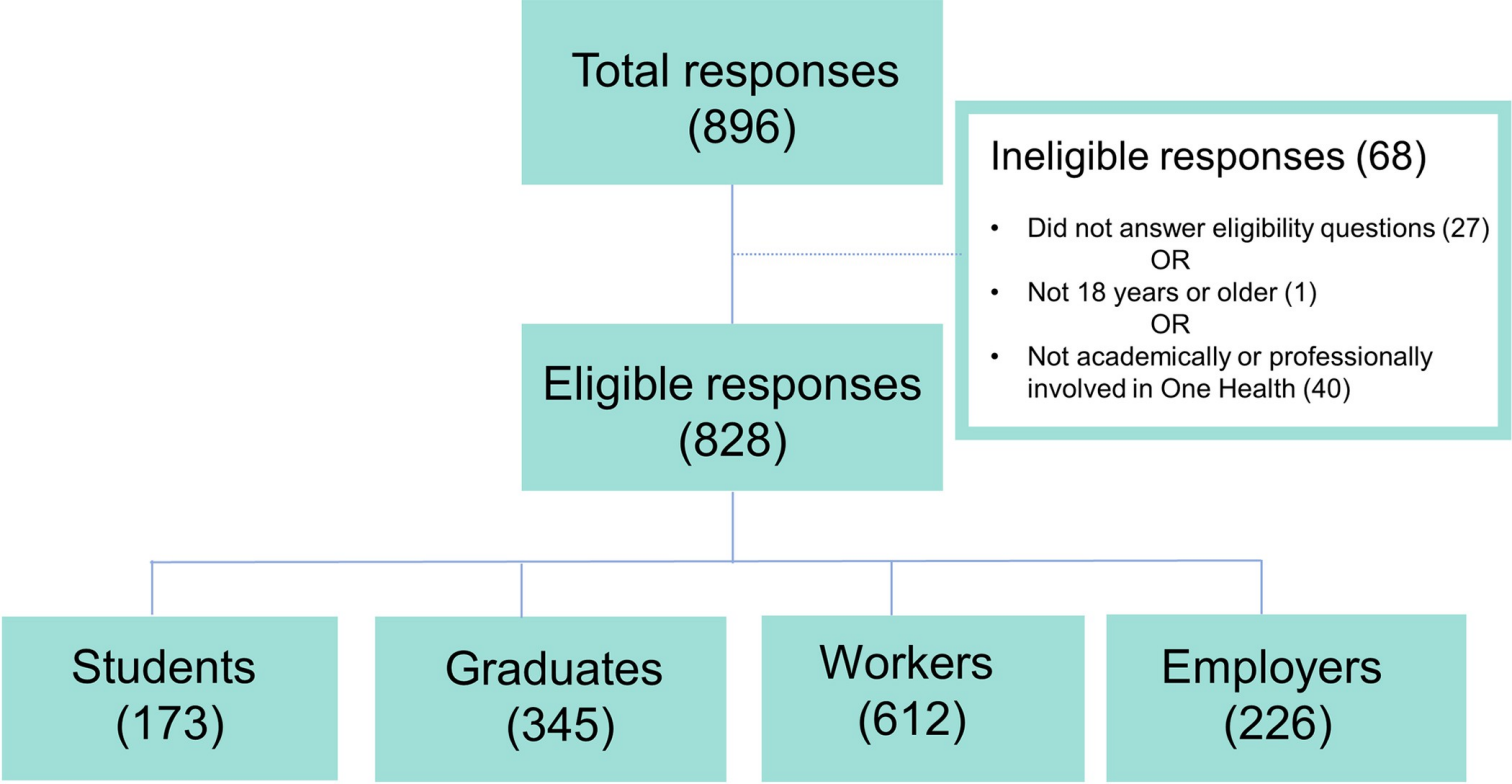
Categorization of total respondents who participated in a multinational One Health workforce survey (2018–2019; n = 828). The total number of students, graduates, workers, and employers in the bottom row do not sum to the number of eligible respondents (n = 828) because respondents were able to provide responses to questions for more than one category (e.g., a trained One Health practitioner might complete modules for both Graduates and Workers).

The survey was disseminated to One Health professional societies and networks, such as the One Health Commission and International Student One Health Alliance, which resulted in posting in online newsletters of these communities; key contacts identified by members of the One Health Action Collaborative; One Health academic programs that had been previously identified [30]; other personal networks of the authors; and organizations identified through a web-based search of employers using a One Health approach. The survey was distributed by email from 16 November 2018 to 1 February 2019 (78 days) with an online survey link (supplementary material). Potential respondents were given an initial offering of the survey and up to two reminders indicating that the survey was open. Respondents were ≥18 years of age and were actively, at the time of the survey, or formerly academically or professionally involved in interdisciplinary work involving human, animal, plant, and/or environmental health. Respondents who started the survey but were under 18 years old, and those who were not involved in interdisciplinary work involving human, animal, plant, and/or environmental health did not proceed to the main section of the survey, so their responses were not included in the analyses (Fig 1).

We collected information on gender, age, highest academic training completed, and current primary country of residence for all respondents (S1 File). For students, the main outcome variables included reasons for pursuing their course of study, emphasis or strength in their program, plans to participate in practical training, the types of organizations in which they hope to secure a position in the future, and plans for further education. For graduates, outcome variables included skills from their One Health education that have been most valuable and how academic training in One Health benefited them in the workplace, as well as the skills they wish had been stronger in their education. For workers, the outcome variables included the most important knowledge or skills they use to carry out their work; method of finding their current positions; and technical, structural, and communication-related challenges they have faced in their job setting. For employers, outcome variables included the number of workers they hired in the past 24 months; desired knowledge, skills, and training they would like their recruits to possess, the platforms they use to recruit workers, and challenges with the recruitment and retention of One Health workers.

A “One Health degree” is defined in this manuscript as a formal educational program granted after completion of high school, including university and college, with a focus on interdisciplinary collaboration among human health, animal health, plant health, and/or environmental health (i.e., One Health) which grants a formal academic degree after completion, excluding certificates. As of January 2023, there was no standardized accreditation system for One Health degree programs. With increasing recognition of the importance of One Health, the number of programs employing the One Health approach is increasing along with the breadth of disciplines involved [30]. While no single organized educational organization or accreditation body could be employed to direct the questionnaire to all One Health graduates, the engagement of numerous One Health professional associations was used to optimize survey responses from this community.

A “One Health worker” is defined in this manuscript as a person who works in a position that requires interdisciplinary collaboration among human health, animal health, plant health, and/or environmental health (i.e., One Health), including full-time or part-time jobs, paid or unpaid internships, externships, and volunteer work. In this survey, participants self-identified as a One Health worker using the definition above. Recognizing that self-identification may not comprehensively capture all workers of interest, we relied on the survey participants’ self-identification because the One Health approach may be employed variably in the workplace, and a centralized registry of the One Health workforce does not exist.

In 2018, updated recommendations for core competencies in One Health education were published [30], and 20 competencies were identified and grouped into three categories; (i) health knowledge; (ii) global and local issues in humans, animals, plants, and the environment; and (iii) professional characteristics. At least three competencies which were assessed to be most relevant and representative of each category were selected by the survey committee, and a total of 13 competencies were included as response options for four questions in the survey (S1 File). Examples of competencies included: development of biosurveillance, diagnostics, and/or therapeutic countermeasures; effective identification of, and relationship among, local and global key stakeholders in One Health; ability to conduct ethical, scientifically sound research that will inform policy.

### Analysis

Survey data was transferred from Qualtrics® to a comma-delimited values file and analyzed using R statistical software version 3.5.1 and Microsoft Excel version 2017. The frequency and percentage of responses were calculated for binary and multiple-choice questions. Free text comments were categorized, analyzed and presented as themes in aggregate.

### Ethics

The project was determined to be exempt by the University of California, Davis, Institutional Review Board (IRB ID: 1312001–1). As per guidance from the Institutional Review Board, a statement about informed consent was included at the beginning of the survey (see S1 File). Individuals participating in the survey were notified that their records would be kept anonymous and confidential; their decision to take part in this survey was completely voluntary; they were free to decline to take part in the project, decline to answer any questions, or stop taking part in the project at any time; whether or not they chose to participate, or answer any question, or stop participating in the project, there would be no penalty to them or loss of benefits to which they were otherwise entitled; and that their return of this survey implied their consent to participate in this survey. No minors (individuals under the age of 18) were eligible to participate in the study.

## Results

### Respondent characterization

**Fig 2:**
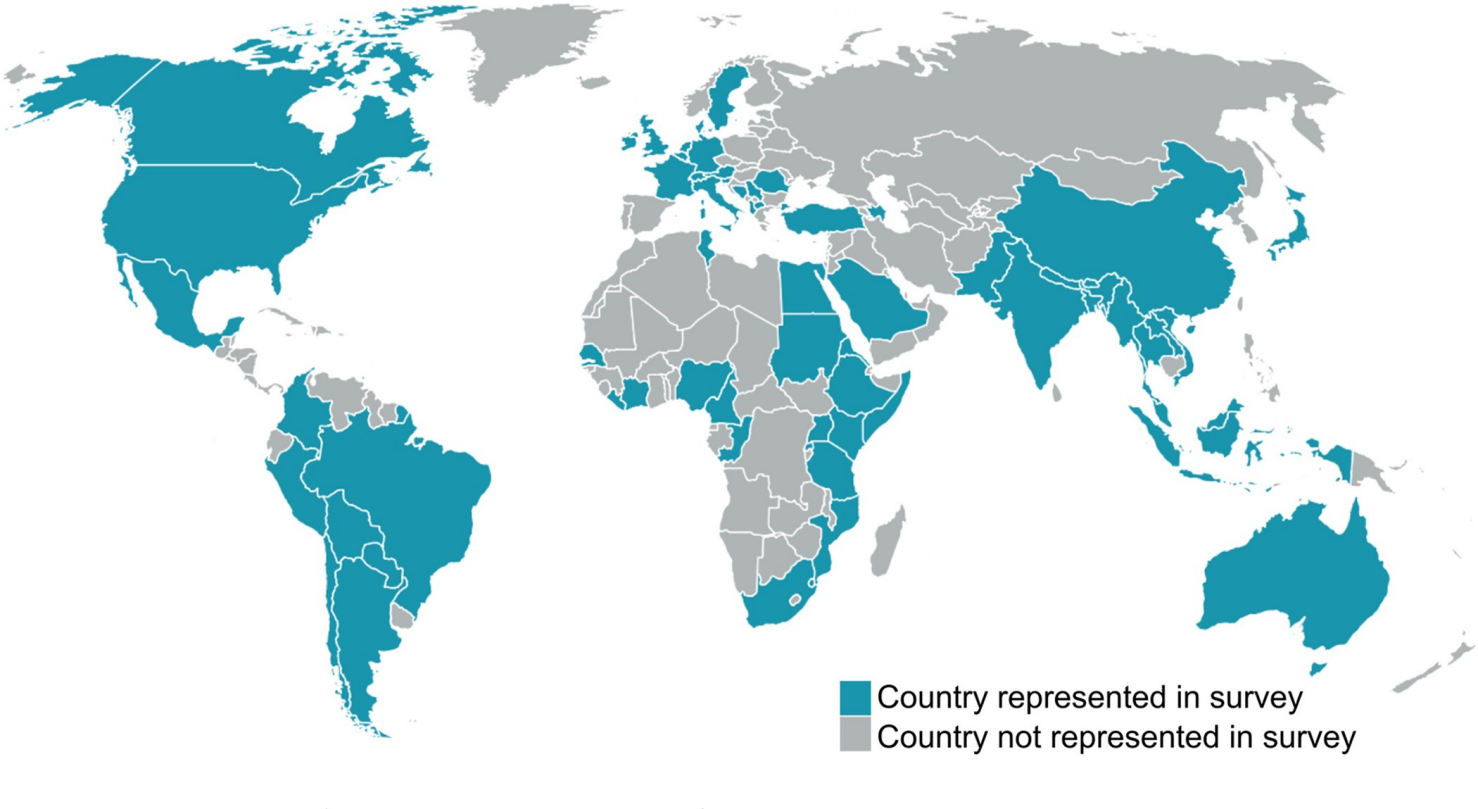
Sixty-six countries represented by 828 respondents to the One Health workforce survey, 2018–2019. The map was generated using Quantum GIS software, version 2.18.27. The shapefile for global administrative boundaries were obtained from Natural Earth (http://www.naturalearthdata.com/). Map date: 11 November 2020, survey dates: 16 November 2018 to 1 February 2019.

A total of 896 responses were recorded from participants representing governmental and non-governmental organizations, academic institutions, professional networks and platforms, student groups, and other relevant stakeholders (Table 1, Fig 2). More than half (57%) of respondents were female, and ages 30 to 39 years were most frequently represented (24%). More than half (56%) of respondents had completed a professional degree in medicine, veterinary medicine, dentistry, or pharmacology. One Health workers and employers were mostly employed by academic institutions, such as universities (42% workers; 44% employers), national or federal government offices (21% workers; 22% employers), and non-governmental or non-profit organizations (15% workers; 17% employers).

**Table 1:** Characteristics of students, graduates, workers, and employers who participated in the One Health workforce survey (n = 828).

	Student (column %)	Graduate (column %)	Worker (column %)	Employer (column %)	Total (column %)
**Count (n)**	**173**	**345**	**612**	**226**	**828**
**Gender**					
Female	108 (62)	206 (60)	355 (58)	111 (49)	469 (57)
Male	63 (36)	134 (39)	251 (41)	111 (49)	321 (39)
Non-binary/third gender	0 (0)	1(<1)	1 (<1)	1 (<1)	2 (<1)
Prefer not to answer	2 (1)	3 (1)	4 (1)	2 (1)	5 (<1)
No response	0 (0)	1 (<1)	1 (<1)	1 (<1)	31 (4)
**Age group (years)**					
18–29	102 (59)	73 (21)	87 (14)	16 (7)	175 (21)
30–39	42 (24)	101 (29)	167 (27)	51 (23)	199 (24)
40–49	23 (13)	76 (22)	141 (23)	57 (25)	175 (21)
50–59	6 (3)	50 (14)	111 (18)	58 (26)	129 (16)
60–69	0 (0)	35 (10)	85 (14)	38 (17)	95 (12)
70–79	0 (0)	6 (2)	16 (3)	4 (2)	19 (2)
80+	0 (0)	1 (<1)	2 (<1)	0 (0)	2 (<1)
Prefer not to answer	0 (0)	3 (1)	3 (1)	2 (1)	4 (<1)
No response	0 (0)	0 (0)	0 (0)	0 (0)	30 (4)
**Degree completed**					
Bachelor’s degree of 4 years or less	82 (47)	175 (27)	309 (50)	104 (46)	391 (47)
Master’s degree (include MPH, MPVM, etc.)	76 (44)	194 (56)	331 (54)	114 (50)	407 (49)
Professional degree in medicine, veterinary medicine, dentistry, or pharmacology	71 (41)	226 (66)	366 (60)	139 (62)	461 (56)
Research-based doctoral degree [Doctor of Philosophy (PhD) or Doctor of Public Health (DrPH)]	30 (17)	127 (37)	246 (40)	112 (50)	287 (27)
Other professional degrees	8 (27)	26 (8)	55 (28)	20 (28)	64 (8)

The number of respondents categorized by regions is summarized in S1 Table.

Column % may not add to 100% due to rounding.

There were 66 countries represented from all eight SDG geographic regions, and 56% lived in the United States at the time of the survey. When categorized by the World Bank’s economic development [31], 582 of 780 respondents with information available on country of residence (75%, 582/780) lived in 22 high income countries (HIC), 150 (19%, 150/780) lived in 36 middle income countries (MIC), and 48 (6%, 48/780) resided in nine low-income countries (LIC).

### Emphases in training and competencies

The top three emphases or strengths of One Health degree programs, in which students were currently enrolled or in which graduates had been previously enrolled, were epidemiology, public health, and zoonoses and emerging infectious diseases (S2 Table). Response options included previously identified key areas represented in One Health degree programs [30] and a free text option. These same three emphases were the highest ranked educational competencies that workers found useful in their current positions (Table 2) and were consistent with the training employers found most desirable in their hires. Training emphases which were desired by employers, but ranked lower in students’, graduates’, and workers’ opinions were professional skills, as previously identified [30], policy, and geographic information systems. Emphases less frequently represented as important across all respondent groups included toxicology, plant health, law, urban planning, and resources and disaster management.

**Table 2:** Important knowledge or skills as defined by graduates, workers, and employers in the One Health workforce survey.

Knowledge or skills*	Graduates		Workers	Employers
	In the past 12 months, what knowledge or skills from your education in One Health do you **most often use** in the workplace? 1	What knowledge and skills from your One Health education have been **most valuable**? 3	What are the most important specific knowledge or skills you currently use to carry out your work in One Health? 3	What are the most important specific knowledge or skills you look for in a One Health worker? 3
	Count (% of n = 345)	Count (% of n = 345)	Count (% of n = 612)	Count (% of n = 226)
Interpersonal communication and communication with scientific or non-scientific audiences	174 (50)	155 (45)	352 (58)	138 (61)
Ability to build, work in, and manage a transdisciplinary team, including addressing conflicts	146 (42)	131 (38)	300 (49)	124 (55)
Scientific principles that influence complex challenges in health (e.g., biological complexity, genetic diversity, interactions of systems, etc.)	102 (30)	130 (38)	204 (33)	86 (38)
Etiology, evolution, and ecology of infectious disease agents	132 (38)	120 (35)	253 (41)	66 (29)
Program / project management	80 (23)	64 (19)	141 (23)	60 (27)
Disease dynamics	115 (33)	117 (34)	230 (38)	55 (24)
Effective identification of, and relationship among local and global key stakeholders in One Health	82 (24)	81 (23)	177 (29)	50 (22)
Ability to conduct ethical, scientifically sound research that will inform policy	56 (16)	82 (24)	113 (18)	49 (22)
Development of biosurveillance, diagnostics, and/or therapeutic countermeasures	71 (21)	69 (20)	134 (22)	48 (21)
Cultural awareness	58 (17)	50 (14)	107 (17)	47 (21)
Cultural and socioeconomic determinants and impacts of illness	55 (16)	63 (18)	102 (17)	35 (15)
Structure and responsibilities of the public health system	56 (16)	45 (13)	102 (17)	32 (14)
Ability to conduct qualitative research to study social and behavioral factors	31 (9)	33 (10)	58 (9)	16 (7)
Other	9 (3)	7 (2)	27 (4)	14 (6)

* Respondents were asked to select up to five options. Knowledge and skills are presented in descending order based on the frequency of employers’ responses.

Students identified the following as study areas they would prefer their degree programs to emphasize more: environmental health, plant health, ecology, wildlife health, field and practical experiences, career development, politics, anthropology, ethics, big data analysis, communication, and team building.

The same top two competencies were used most frequently by graduates and most valued by graduates, workers, and employers:

- Interpersonal communication and communication with scientific or non-scientific audiences
- Ability to build, work in, and manage a transdisciplinary team, including addressing conflicts.

One employer responded that the “(lack of) practical skill(s) on interpersonal communication” in their potential hires was a challenge when recruiting employees, highlighting the importance of communication skills in the workplace. Competencies in interpersonal communication are highly valued in One Health, especially because experts with diverse backgrounds often work collaboratively in a transdisciplinary team. As an example, one worker identified “developing multidisciplinary partnerships, (and) developing goals and objectives that appeal to a multidisciplinary group” as the type of training they would have liked to have had that would benefit them in conducting their current work.

Other competencies perceived as important by graduates, workers, and employers were:

- Scientific principles that influence complex challenges in health (e.g., biological complexity, genetic diversity, interactions of systems, etc.)
- Etiology, evolution, and ecology of infectious disease agents
- Understanding of disease dynamics
- Effective identification of, and relationship among, local and global key stakeholders in One Health
- Ability to conduct ethical, scientifically sound research that will inform policy
- Development of biosurveillance diagnostics and/or therapeutic countermeasures

Employers ranked program management as the fifth most important core competency desirable in a One Health worker, which was a higher rank than that of graduates and workers (Table 2). For example, employers indicated the need for “…staff with deep specific technical and leadership/management skills who understand the necessity of taking One Health approaches in all they do”, while highlighting challenges to “find potential candidates who possess technical skills and experience, but are willing to apply them to broader program management”.

### Reasons to pursue higher education in One Health

Students reported becoming interested in One Health through information from their mentor or advisor (54% of students who responded 80/147; 52 from HIC, 23 from MIC, 4 from LIC, 1 with no country information); meetings or conferences (48%, 70/147; 44 from HIC, 20 from MIC, 3 from LIC, 3 with no country information); and reading journals, books, and other texts (47%, 69/147; 46 from HIC, 16 from MIC, 5 from LIM, 2 with no country information). These trainees indicated that they decided to pursue their course of study with a focus on One Health to make a more significant contribution to health (86%, 125/146; 81 from HIC, 34 from MIC, 8 from LIC, 2 with no country information), develop professional skills or perform better in their jobs (64%, 93/146; 53 from HIC, 31 from MIC, 6 from LIC, 3 with no country information), or pursue research interest (60%, 87/146; 54 from HIC,28 from MIC, 4 from LIC, 1 with no country information). Graduates ranked the top three reasons to pursue their study in the same order as students.

The most important factors students ranked for selecting their degree programs were course curriculum and research focus (79%, 111/141; 67 from HIC, 32 from MIC, 9 from LIC, 3 with no country information), followed by reputation of academic institution or degree program (53%, 75/141; 50 from HIC, 18 MIC, 4 from LIC, 3 with no country information) and geographic area (38%, 53/141; 36 from HIC, 11 from MIC, 4 from LIC, 2 with no country information). The least important factor was the presence or absence of a qualifying exam or thesis (8%, 11/141; 7 from HIC, 3 MIC, 1 with no country information).

Among degree programs with which respondents were affiliated, 61% (88/145; 50 from HIC, 27 from MIC, 8 from MIC, 3 with no country information) of students’ and 55% (153/279; 107 from HIC, 32 from MIC, 11 from LIC, 3 with no country information of graduates’ programs required them to participate in practical training experiences, internships, or externships. Irrespective of program requirement, 90% (129/143; 76 from HIC, 39 from MIC, 10 from LIC, 4 with no country information) of students and 61% (88/145; 50 from HIC, 27 from MIC, 8 from LIC, 3 with no country information) of graduates already participated, or were planning to participate, in practical training. Almost all students (91%, 108/111; 60 from HIC, 39 from MIC, 7 from LIC, 2 with no country information) and graduates (97%, 171/177; 121 from HIC, 34 from MIC, 14 from LIC, 2 with no country information) who participated in a practical experience found it to be helpful. Conversely, the reasons given for why practical experiences were unhelpful included poor structure of experience and organization, limited hands-on experience, or that activities did not match with student interest.

The majority of students (71%, 102/144; 52 from HIC, 36 from MIC, 10 from LIC, 4 with no country information) planned to pursue further education beyond their current course of study, such as a research-based doctoral degree (28% 40/144; 22 from HIC, 14 from MIC, 2 from LIC, 2 with no country information); a professional degree in medicine, veterinary medicine, dentistry, or pharmacology (23%, 33/144; 22 from HIC, 6 from MIC, 4 from LIC, 1 with no country information); or a Master’s degree (22%, 32/144; 16 from HIC, 9 from MIC, 4 from LIC, 3 with no country information). Among 56 students, whose highest level of education was a bachelor’s degree of four years or less, 43% (24/56, 15 from HIC, 5 from MIC, 2 from LIC, 2 with no country information) wished to pursue higher education in a Master’s degree; and/or a professional degree in medicine, veterinary medicine, dentistry or pharmacology (36%, 20/56, 15 from HIC, 1 from MIC, 3 from LIC, 1 with no country information); and/or a research based doctoral degree (20%, 11/56, 7 from HIC, 2 from MIC, 1 from LIC, 1 with no country information), while 9% (5/56, 4 from HIC, 1 from MIC) were undecided and 7% (4/56, all 4 from HIC) did not plan to pursue further education. Most students (80%, 116/145; 66 from HIC, 39 from MIC, 9 from LIC, 2 with no country information) stated that they hope to work on challenges that affect countries other than their country of origin or schooling.

The majority (85%, 468/553, 367 from HIC, 75 from MIC, 17 from LIC, 9 with no country information) of One Health workers were aware of academic degree programs focusing on One Health offered at the undergraduate, Master’s, and doctoral levels. Over half (57%, 319/556; 244 from HIC, 53 from MIC, 18 from LIC) of workers had received training in One Health at the undergraduate or graduate level, such as taking a course covering topics related to One Health or attending a program with a One Health approach.

When One Health employers were asked if they believed that the current One Health programs have been producing graduates that meet their needs, 16% (30/190; 20 from HIC, 8 from MIC, 2 from LIC) responded “yes”; 19% (36/190; 27 from HIC, 4 from MIC, 2 from LIC, 3 with no country information) responded “no”; and 65% (124/190; 102 from HIC, 14 from MIC, 6 from LIC, 2 with no country information) were unsure. Some examples of degree programs that employers mentioned had produced graduates that meet their needs are listed in S3 Table.

### Work and employment

More than half (75%, 139/186; 122 from HIC, 9 from MIC, 3 from LIC, 5 with no country information) of employers used online public job postings to recruit One Health workers, whereas only 27% (146/545; 118 from HIC, 18 from MIC, 5 from LIC, 5 with no country information) of One Health workers found their current positions online. In the 24 months prior to taking the survey, 50% (91/183; 74 from HIC, 12 from MIC, 3 from LIC, 2 with no country information) of employers had hired one to three One Health workers, 33% (60/183; 47 from HIC, 10 from MIC, 2 from LIC, 1 with no country information) did not hire any, and 6% (11/183; 6 from HIC, 1 from MIC, 3 from LIC, 1 with no country information) hired 15 or more workers [range 0 to 16+]. Among current One Health workers, 29% (154/527; 116 from HIC, 30 from MIC, 6 from LIC, 2 with no country information) faced challenges finding their current position. As for employers, 31% (55/176; 44 from HIC, 10 from MIC, 1 from LIC) had challenges recruiting One Health workers, and 27% (47/176; 34 from HIC, 11 from MIC, 2 from LIC) had challenges retaining them. Although 56% (80/143; 45 from HIC, 27 from MIC, 7 from LIC, 1 with no country information) of students expressed hopes of finding a position in international organizations, only 9% (49/555, 29 from HIC, 16 from MIC, 4 from LIC) of workers and 9% (19/210, 6 from HIC, 10 from MIC, 2 from LIC) of employers were based in international organizations.

Among employers who indicated that they had difficulty recruiting One Health workers and explained the reason for this challenge, 33% (15/46, 12 from HIC, 3 from MIC) indicated that there was a limited pool of candidates who were qualified, suitable, or adequately prepared for the desired position. Some employers stated that “a good, motivated member is always a missing need”, “there aren’t enough of them (the right candidates)” and “finding individuals wanting to work outside their core discipline”. Among workers who had difficulty finding a position in One Health, 18% (24/134, 22 from HIC, 2 from MIC) indicated limited numbers and availability of positions. One worker said that “There were hardly any One Health related positions for an early career professional". Workers and employers who indicated that they had difficulty finding or recruiting for jobs were distributed in multiple countries and regions. Some workers identified challenges for unclear career pathways for people trained in One Health: “I suspect that there is limited knowledge in the public health sector that there is even the possibility of a career in One Health” and “(there is) no clear career path”.

Among 123 employers (98 from HIC, 14 from MIC, 7 from LIC, 4 with no country information) who had hired one or more One Health workers within 24 months of responding to the survey, 55% (68/123, 58 from HIC, 7 from MIC, 1 from LIC, 2 with no country information) did not hire any employees who held a One Health degree. When asked if they planned to hire more One Health workers in the future, employers responded “yes” (53%, 102/194; 80 from HIC, 15 from MIC, 6 from LIC, 1 with no country information), “maybe” (43%, 83/194; 66 from HIC, 10 from MIC, 3 from LIC, 4 with no country information), and “no” (5%, 9/194; 8 from HIC, 1 from MIC).

When asked if employers experienced challenges in retaining One Health workers, 27% (47/176, 34 from HIC, 11 from MIC, 2 from LIC) responded “yes”. The most common challenge cited by these employers in retaining One Health workers was financial, such as those related to funding availability and consistency and salary competitiveness (46%, 17/37; 10 from HIC, 7 from MIC). One employer indicated that there was a “lack of dedicated resources at the federal level”, and one worker pointed to the “lack of dedicated One Health budget lines to ensure a sustainable workforce” as a challenge with employing or retaining One Health workers in the workplace. Other challenges included limited career opportunities and low salary competitiveness compared to other employers and sectors (11%, 4/37; 3 from HIC, 1 from LIC), limited availability of long-term, stable positions with career advancement opportunities (8%, 3/37; 3 from HIC), employees leaving position for further education or career advancement (5%, 2/37; 2 from HIC), and ill-defined career trajectories for One Health workers (3%, 1/37 from HIC). One worker identified “frustration with lack of consistent definition and lack of value placed on One Health” as possible challenges in retaining One Health workers.

## Discussion

The impact of COVID-19 on health, society, and the economy highlights the criticality of One Health competencies for addressing infectious diseases, food insecurity, and responding to natural disasters and extreme weather events including those rooted in climate change. The emergence of health risks, including climate change-induced migration, population displacement, conflict, and known and emerging infectious pathogens underscore the importance of collaborative efforts between health practitioners and policy makers [32, 33]. It is all the more important for health equity because the health burden is disproportionately placed on vulnerable populations including low-income and underrepresented countries and communities [3]. The One Health workforce are expected to be able to better assess the risk of health emergencies by recognizing the interconnectedness among hazards, affected populations, and the environment. They can also facilitate information sharing across sectors and disciplines. Our survey was unique in that it went beyond capturing the number of individuals trained and has collected the lived experiences of the One Health workforce. While it is challenging to characterize a workforce that is not officially certified or centrally registered, this study included 828 respondents who represented 66 countries, age groups 18–29 years to >80 years, as well as individuals across career stages. This is the first study to capture perspectives of One Health workers with this level of geographic and demographic representation.

Demographic data on the One Health workforce is absent. Therefore, we compared our findings about gender with specific health sectors whose workforce is much better characterized. For example, 70% of the global health and social care workforce are women, although only 25% hold senior roles according to WHO [34]; 36% of active physicians in the United States (US) are female, according to the Association of American Medical Colleges [35] which is consistent with WHO data that women comprised less than half of the physician workforce in five of six WHO Regions [36]. Conversely, in nursing, females accounted for 65% or more of the workforce in all six WHO Regions [36]. In veterinary medicine in 2019, 63% of the US veterinary workforce were women [37]. In dentistry in 2016–2018, the proportion of women who were registered dentists varied across countries in Oceania, Asia, Europe, Africa and Latin America with available data, ranging from 32% in Hong Kong Special Administrative Region and the Republic of Korea to 70% in India [38]. The variability of the proportion of female health workers is high, and the proportion varies across discipline and countries. This information justifies the need for follow up studies to better characterize the One Health workforce.

Funding limitations and lack of availability of long-term, stable positions with career advancement opportunities most commonly challenged employers to retain One Health employees. Understanding competencies that support a One Health worker in the workplace will allow training programs to better prepare individuals to be successful and thrive in the workplace. Additionally, identifying employment challenges, as we have, should inform and help funders and employers identify, recruit and retain quality One Health workers with the highest potential for success.

Successful interdisciplinary and multisectoral projects require consistent coordination to deliver results on time, in budget, and to scope. Therefore, a combination of interpersonal skills and scientific knowledge to understand complex health challenges support the successful worker in the workplace [39–41]. A previous study endorsed the importance of communication and proficiency in at least one area of health science in One Health training [30]. Our findings further emphasize that a combination of interpersonal skills, discipline-specific knowledge, and practical application supports a successful One Health worker. While graduates and workers placed relatively low importance on program management, employers valued the skill in an employee. A clear outline of what employers can expect in employing a One Health worker is required. We believe that employers can expect the following three competencies when hiring a worker with One Health training, ability to: (i) demonstrate and exercise a One Health approach to solve complex challenges related to health and sustainable development, regardless of the sector or discipline with which the worker is affiliated; (ii) take leadership and proactively seek opportunities to work across disciplines and organizations, recognizing the benefits of interdisciplinary collaboration; and (iii) build, work in, and/or lead a collaborative team, and contribute disciplinary expertise in the collaborative team.

A misalignment in One Health terminology, definitions, and expectations regarding job postings, titles, and ultimate responsibilities of the job seeker may be hindering the matching of job seekers and employers. One possible explanation is that job postings, which more than half of employers use for recruitment, are not being seen by the best qualified applicants. Another possible reason is that job seekers may be looking for the term “One Health” in the title or summary of responsibilities, rather than understanding how their One Health skills apply to a wide range of jobs. Students and graduates will likely identify more opportunities if they seek out positions that do not explicitly indicate “One Health” in the job title or description, but instead feature concepts or opportunities to apply One Health skills (Table 3). For employers, we encourage using the term “One Health” and associated competencies in job descriptions. Addressing the disconnect between job seekers and employers, such as through an improved platform for One Health related opportunities, is an opportunity for maximizing the application of competencies acquired in educational programs to the workplace.

**Table 3:** Recommendations for students and early-career professionals, workers, and employers in One Health.

**Students and early-career professionals**	• Speak with and learn from professionals and mentors already working in your field of interest in One Health, because there are many ways to work in One Health; • Evaluate how you would like to engage in One Health and what expertise you would like to bring to an interdisciplinary or multisectoral team; • When seeking a job, understand that the One Health approach is essential to most jobs within the relevant sectors, even if an opportunity is not explicitly advertised as “One Health”. If possible, design work with a One Health scope and apply skills to add needed value and perspective; • Build program leadership and management skills through practical experiences and coursework; • Recognize that local and regional applications of One Health are as important as international ones; • Become involved in professional societies to build relationships, establish your collaborative network, hone your skills, and become more involved in your field of interest.
**Workers**	• Make sure all team members understand roles and responsibilities of members from other disciplines and sectors; • Be proactive in taking on new assignments and opportunities to use your One Health competencies; • Seek opportunities to work across disciplines and organizations, and demonstrate your collaborative, team building skills; • Avoid the use of jargon or technical terms when communicating in an interdisciplinary or multisectoral team; • Make an effort to understand your team members’ expertise, skillsets, and baseline knowledge of subject matter; • Assign roles and responsibilities early on in projects, as is important in any professional situation; • Describe in detail to employers how a One Health approach can enhance the organization’s goals; • Use consistent language around the meaning of One Health to increase awareness and understanding; • Present and publish on your work at meetings, events, etc. outside of your discipline and sector to expand One Health networks and partnerships.
**Employers**	• Hire candidates with an interdisciplinary and collaborative approach, even for positions which do not include “One Health” in the position title; • Explicitly use the term “One Health” and associated skills and competencies in job descriptions to help applicants identify positions as appropriate for their submission; • Publicly share career trajectories and experiences of professionals working in your institutions; • Ask current employees and candidates how One Health concepts can be integrated into your organization; • Encourage workers to grow on the job, expand their value through continuing education and learning opportunities in One Health; • Host students/interns or mentor students to help increase experience in One Health areas and promote One Health through student channels.

The release of “A Tripartite Guide to Addressing Zoonotic Diseases in Countries” [42] emphasizes the benefits of fully embracing and adopting the transdisciplinary and multisectoral One Health approach. To date, One Health has developed and gained support mainly in its application for addressing zoonoses, as seen in the programmatic emphases (zoonoses, public health, and epidemiology) listed by our respondents. Building on these successes, One Health now encompasses a broader range of topics, including food security and safety, vector-borne diseases, climate variability, and antimicrobial resistance. One Health workers can also understand the drivers of complex issues and implement effective interventions in other disciplines, including social and behavioral sciences, information technology, plant health, and ecology [4, 43]. Using a clear and consistent definition that can apply to a broad range of challenges is important to better understand the value of One Health. We endorse the following working definition used by the United States federal agencies–One Health is defined as “a collaborative, multisectoral, and transdisciplinary approach–working at the local, regional, national, and global levels–with the goal of achieving optimal health outcomes recognizing the interconnection between people, animals, plants, and their shared environment” [7].

The multitude of settings in which a worker can thrive in One Health enables a flexible and exciting career, though at times, career trajectories are difficult to define. The lack of understanding or awareness of One Health competencies or applicability to jobs may impact retention. Employee turnover is costly to institutions and hinders team productivity and effectiveness, and retaining competent workers is a ubiquitous challenge that employers and countries face [12]. Employee retention is especially important to sustain health care systems and address the shortage of health care professionals, in light of increased resignation and reports of health care workers’ mental health being impacted by COVID-19 [44]. Close cooperation between health employers and occupational health professionals could improve the overall well-being of One Health workers and may increase their retention in the workplace [45]. As our survey found, ill-defined career trajectories, challenges in career advancement, and limited availability of stable positions hinder the retention of One Health workers. One Health workers could share success stories of their career path and advancement to foster positive decision-making individuals at all stages of career development. We also encourage current One Health workers to provide opportunities to students by hosting student interns and/or mentoring students through practical training positions (Table 3).

These findings, while comprehensive, should be interpreted with consideration of their limitations. First, the respondents of this survey are likely not representative of the entire workforce that employs a One Health approach, as the sample is based on volunteer respondents and does not encompass the entire workforce trained in One Health. A majority of our participants are from high-income countries, so the perspectives captured in the study more readily reflect experiences of people from high-income countries as compared to people from middle- and low-income countries. Indeed, while we attempted to reach out through all possible One Health networks, there is no single association through which we could recruit participants. In addition, while many people have been trained in One Health through academic programs designed to fill the employment gap, many graduates may not be practicing One Health after graduation and thus may not have seen the solicitation for participation through the professional networks used.

Second, the cross-sectional study design did not allow for us to capture temporal changes in the respondents’ perspectives. We collected survey data in late 2018 and early 2019. Our results are therefore valuable records of characteristics of the workforce and challenges in One Health before the COVID-19 pandemic. Similar surveys could be periodically conducted in the future to better understand the changes in perspectives of individuals employing the One Health approach, especially as it is now being widely advocated as a method to improve pandemic preparedness and response [8, 46].

Broadening the understanding of One Health, how it is an approach that is fundamental to many jobs, will open more opportunities for individuals who wish to build a transdisciplinary health career. Recently, the number of One Health academic degree programs have increased, with over 45 One Health degree programs existing in the United States alone, of which 83% were established after 2002 [30]. Reflecting the relevance, societal needs, and popularity of such programs, over half of workers who participated in this survey received training in One Health at the undergraduate or graduate level. As the number of graduates from these educational programs increases and more professionals embrace the One Health approach, the interdisciplinary workforce will continue to grow. We will truly achieve availability of a One Health workforce when the majority of workers in each relevant sector can apply collaborative and transdisciplinary competencies, regardless of title or label.

COVID-19 heightened the interest and highlighted the relevance of the One Health approach. We support employing the One Health approach for a diverse range of positions, even if they do not explicitly include “One Health” in the job title, and using interpersonal communication skills to clarify the expectations, roles and responsibilities within a transdisciplinary team. Collectively, this shift to developing an interdisciplinary and multisectoral global health workforce will enable more substantive progress on SDGs, improve global health security, and prevent or mitigate adverse consequences of health emergencies. Employing a holistic approach will also help develop strategies for resilient and sustainable post-emergency recovery.

For recommendations for educators, please see “Core Competencies in One Health Education: What are we missing?” [30].

## Supporting information

S1 Table Geographic distribution of respondents in 66 countries by regions classified from the United Nations Sustainable Development Goals. (DOCX)

S2 Table Rank of emphases or strengths of One Health degree programs according to students and graduates, and useful foci according to workers and employers, based on the frequency of selection by participants. (DOCX)

S3 Table List of programs and universities that employers listed as producing graduates in One Health that meet their needs, in alphabetical order of country. (DOCX)

S1 File Multinational online survey of the One Health workforce, November 2018 to February 2019. (DOCX)
