# Supplementary material for: Characterizing the One Health workforce to promote interdisciplinary, multisectoral approaches in global health problem-solving
Source: PLoS One. 2023 May 16;18(5):e0285705. doi: 10.1371/journal.pone.0285705 (PMC10187933; doi:10.1371/journal.pone.0285705)
Supplement: S1 Table — (DOCX) [file pone.0285705.s001.docx]

**Supplementary information**

**S1 Table. Geographic distribution of respondents in 66 countries by regions classified from the United Nations Sustainable Development Goals.**

| **SDG regions and countries** | **Count** | **%** | **SDG regions and countries** | | **Count** | **%** |
| --- | --- | --- | --- | --- | --- | --- |
| **Northern America** | **498** | **60·1** | **Eastern and South-Eastern Asia** | | **48** | **5·8** |
| United States of America | 467 | 56**·**4 | Malaysia | | 19 | 2·3 |
| Canada | 31 | 3**·**7 | Thailand | | 13 | 1·6 |
| **Sub-Saharan Africa** | **93** | **11.2** | Japan | | 4 | 0·5 |
| Ethiopia | 17 | 2.1 | Hong Kong, Special Administrative Region | | 3 | 0·4 |
| Kenya | 16 | 1.9 | Indonesia | | 2 | 0·2 |
| Nigeria | 12 | 1**·**4 | Viet Nam | | 2 | 0·2 |
| Cameroon | 13 | 1.6 | China | | 1 | 0·1 |
| Tanzania | 9 | 1**·**1 | Lao PDR | | 1 | 0·1 |
| Rwanda | 6 | 0**·**7 | Myanmar | | 1 | 0·1 |
| Uganda | 5 | 0**·**6 | **Latin America and the Caribbean** | | **38** | **4·6** |
| Senegal | 3 | 0**·**4 | Brazil | | 11 | 1·3 |
| Sudan | 3 | 0**·**4 | Argentina | | 8 | 1·0 |
| South Africa | 2 | 0**·**2 | Chile | | 6 | 0·7 |
| Eritrea | 1 | 0·1 | Saint Kitts and Nevis | | 3 | 0·4 |
| Republic of Congo | 1 | 0·1 | Colombia | | 3 | 0·4 |
| Cote d’Ivoire | 1 | 0·1 | Trinidad and Tobago | | 2 | 0·2 |
| Liberia | 1 | 0·1 | Bolivia | | 2 | 0·2 |
| Mozambique | 1 | 0·1 | Mexico | | 1 | 0·1 |
| Somalia | 1 | 0·1 | Paraguay | | 1 | 0·1 |
| Swaziland | 1 | 0·1 | Peru | | 1 | 0·1 |
| **Europe** | **64** | **7.7** | **Central and Southern Asia** | | **24** | **2·9** |
| United Kingdom | 12 | 1·4 | India | | 7 | 0·8 |
| France | 10 | 1·2 | Bangladesh | | 7 | 0·8 |
| Switzerland | 9 | 1·1 | Nepal | | 7 | 0·8 |
| Belgium | 5 | 0·6 | Pakistan | | 2 | 0·2 |
| Germany | 5 | 0·6 | Bhutan | | 1 | 0·1 |
| Ireland | 4 | 0·5 | **Northern Africa and Western Asia** | | **7** | **0.8** |
| Italy | 4 | 0·5 | Azerbaijan | | 2 | 0·2 |
| Bosnia and Herzegovina | 3 | 0·4 | Turkey | | 2 | 0·2 |
| Austria | 2 | 0·2 | Egypt | | 1 | 0·1 |
| Netherlands | 2 | 0·2 | Saudi Arabia | | 1 | 0·1 |
| Albania | 1 | 0·1 | Tunisia | | 1 | 0·1 |
| Denmark | 1 | 0·1 | **Oceania** | | **8** | **1·0** |
| Malta | 1 | 0·1 | Australia | | 8 | 1·0 |
| Macedonia | 1 | 0·1 | Missing | 58 | | 7.0 |
| Serbia | 1 | 0·1 |  |  |  |  |
| Romania | 1 | 0·1 |  |  |  |  |
| Sweden | 1 | 0·1 |  |  |  |  |
| Slovenia | 1 | 0·1 |  |  |  |  |
