# Supplementary material for: Characterizing the One Health workforce to promote interdisciplinary, multisectoral approaches in global health problem-solving
Source: PLoS One. 2023 May 16;18(5):e0285705. doi: 10.1371/journal.pone.0285705 (PMC10187933; doi:10.1371/journal.pone.0285705)
Supplement: S2 Table — (DOCX) [file pone.0285705.s002.docx]

**Supplementary information**

**S2 Table. Rank of emphases or strengths of One Health degree programs according to students and graduates, and useful foci according to workers and employers, based on the frequency of selection by participants.**

| **Section** | **Students** | **Graduates** | **Workers** | **Employers** |
| --- | --- | --- | --- | --- |
| Question | Is there a specific emphasis or strength in your program? | Was there a specific emphasis or strength in your program? | What did you find most useful for your current position in your One Health training? | What types of training would you like your hires to have? |
| Epidemiology | **2** | **2** | **1** | **1** |
| Zoonoses, emerging infectious diseases | **1** | **1** | **2** | **2** |
| Public health | **3** | **3** | **3** | **3** |
| Environmental health, ecology | 7 | 7 | **4** | **4** |
| Professional skills | 13 | 13 | 13 | **5** |
| Food safety, food security | **5** | 6 | 6 | 6 |
| Antimicrobial resistance (AMR) | 6 | 9 | 9 | 7 |
| Agriculture, livestock | **4** | **4** | **5** | 8 |
| Policy | 14 | 12 | 14 | 9 |
| Geography, Geographic Information Systems (GIS) | 16 | 14 | 15 | 10 |
| Conservation, wildlife | 8 | 8 | 7 | 10 |
| Preventive medicine | 9 | **5** | 8 | 12 |
| Entomology, vector-borne diseases | 9 | 10 | 10 | 13 |
| Molecular biology, genetics & synthetic biology | 11 | 11 | 12 | 13 |
| Social and behavioral sciences | 15 | 16 | 13 | 15 |
| Economics | 20 | 18 | 16 | 16 |
| Qualitative research | 12 | 15 | 18 | 17 |
| Urban planning, resources management & disaster management | 21 | 20 | 20 | 19 |
| Law | 21 | 20 | 23 | 20 |
| Plant health | 18 | 22 | 21 | 21 |
| Toxicology | 16 | 17 | 16 | 21 |
| No specific emphasis | 23 | 23 | 22 | 23 |
| Other | 19 | 19 | 19 | 18 |
