# Supplementary material for: Characterizing the One Health workforce to promote interdisciplinary, multisectoral approaches in global health problem-solving
Source: PLoS One. 2023 May 16;18(5):e0285705. doi: 10.1371/journal.pone.0285705 (PMC10187933; doi:10.1371/journal.pone.0285705)
Supplement: S3 Table — (DOCX) [file pone.0285705.s003.docx]

**Supplementary information**

**S3 Table. List of programs and universities that employers listed as producing graduates in One Health that meet their needs, in alphabetical order of country.**

| **University** | **Degree** | **Country** |
| --- | --- | --- |
| Toulouse University, Paul Sabatier University (Toulouse III) & Agricultural Research for Development (CIRAD) | Master Program, Gestion Integree des Maladies Animales Tropicales (GIMAT) | France |
| Kerala Veterinary University | Unspecified | India |
| University of Nairobi | Bachelor of Veterinary Medicine | Kenya |
| Nigeria Centre for Disease Control | Master of Public Health in Field Epidemiology, Nigerian Field Epidemiology and Laboratory Training Program (NFELTP) | Nigeria |
| Sokoine University of Agriculture | Bachelor of Veterinary Medicine | Tanzania |
| Kasetsart University & Toulouse University | Msc InterRisk in double diploma | Thailand & France |
| Makarere University | Bachelor of Veterinary Medicine | Uganda |
| Royal Veterinary College (RVC) & London School of Tropical Medicine and Hygiene (LSTMH) | MSc in One Health | UK |
| London School of Tropical Medicine and Hygiene | Unspecified | UK |
| Royal Veterinary College | Unspecified | UK |
| University of California, Davis | Master of Preventive Veterinary Medicine, PhD programs | USA |
| University of Arizona | MPH in One Health | USA |
| Ohio State University | Unspecified | USA |
| Duke University | ‘One Health programs’ | USA |
| University of Florida | ‘One Health programs’  MS and PhD | USA |
| Unspecified | MSc in One Health | Unspecified |
