# Supplementary material for: Characterizing the One Health workforce to promote interdisciplinary, multisectoral approaches in global health problem-solving
Source: PLoS One. 2023 May 16;18(5):e0285705. doi: 10.1371/journal.pone.0285705 (PMC10187933; doi:10.1371/journal.pone.0285705)
Supplement: S1 File — (DOCX) [file pone.0285705.s004.docx]

**Supplementary information**

**S1 File. Multinational online survey of the One Health workforce, November 2018 to February 2019.**

[Objectives]

Objectives of this survey are to:

-Characterize students, graduates, workers, and employers in the One Health arena; and

-Understand the benefits of One Health education; and

-Elucidate unique challenges that One Health workers face; and

-Assess whether employers are satisfied with the skillsets of employees who have received training in the One Health arena.

[Definition]

In this survey, One Health is defined as "a collaborative, multisectoral, and transdisciplinary approach – working at the local, regional, national, and global levels – with the goal of achieving optimal health outcomes recognizing the interconnection between people, animals, plants, and their shared environment".

[Consent]

You are being invited to join a survey as part of a study. You are eligible for this survey only if you are 18 years or older. If you agree to participate in this study, you will be asked to answer questions online and submit your responses electronically. Your records will be kept anonymous and confidential. Your decision to take part in this survey is completely voluntary, and you are free to decline to take part in the project, decline to answer any questions, or stop taking part in the project at any time. Whether or not you choose to participate, or answer any question, or stop participating in the project, there will be no penalty to you or loss of benefits to which you are otherwise entitled. If you have any questions about this research, please feel free to contact the investigator. Your return of this survey implies your consent to participate in this survey.

Thank you very much for your participation.

2 Are you 18 years or older?

Yes / No

3 Are you currently or have been in the past involved in the One Health arena academically or professionally? One Health is "a collaborative, multisectoral, and transdisciplinary approach – working at the

local, regional, national, and global levels – with the goal of achieving optimal health outcomes recognizing the interconnection between people, animals, plants, and their shared environment".

Yes / No

4 Please indicate your gender.

Female

Male

Non-binary/third gender

Prefer not to answer

5 Please indicate your age group.

18 to 29 years old

30 to 39 years old

40 to 49 years old

50 to 59 years old

60 to 69 years old

70 to 79 years old

80 years or older

Prefer not to answer

6 What academic training have you completed? Select all that apply. You may choose to specify the name of your degree in the text box if you wish.

Secondary school / high school

Bachelor's degree of 4 years or less

Master's degree (include MPH, MPVM, etc.)

Professional degree in medicine, veterinary medicine, dentistry, or pharmacology

Other professional degrees

Research based doctoral degree [Doctor of Philosophy (PhD)]

Other

7 In which country do you currently live? If you live in more than one country, choose one primary residence.

▼ Afghanistan (1) ... Zimbabwe (195)

8 Are you currently a student in a post-secondary academic degree program with a focus on interdisciplinary collaboration among human health, animal health, plant health, and/or environmental health (i.e., One Health)? Definition of post-secondary: formal education granted after completion of high school, including university and college. Definition of degree program: grants a formal academic degree after completion, excluding certificates.

Yes (1)

No (2)

Start of Block: Section 2: Current students in One Health degree programs

9 In which country are you currently studying? If you study in more than one country, choose one primary country where you spend the majority of your time.

▼ Afghanistan (1) ... Zimbabwe (195)

10 How did you become interested in One Health? Select all that apply.

Internet search

Information from mentor or adviser

Fellow students

Workplace

Meetings or conferences

Reading journals, books, and other texts

Other

11 Why did you decide to pursue your current course of study which requires interdisciplinary collaboration among human, animal health, plant health, and/or environmental health (i.e. One Health)? Select all that apply.

Develop professional skills or perform better in job (2)

Change or advance career (1)

Make a more significant contribution to health (4)

Pursue research interest (5)

Increase possibility for better salary and benefits (7)

Recommended by colleagues (6)

Recruited by faculty (8)

Other (9)

12 What attracted you to the academic degree program with a focus on One Health that you are currently attending? Select the top three options that were most important to you.

Course curriculum and research focus

Presence/absence of qualifying exam or thesis

Faculty members

Alumni network

Geographic area

Tuition and funding availability

Time-to-completion of degree program

Size of program

Reputation of academic institution or degree program

Recommendations from others

Other

13 Is there a specific emphasis or strength in your program? Please base your response on how your program is structured or how you have individually designed your course of study. Select all that apply.

Epidemiology (1)

Environmental health, ecology (2)

Food safety, food security (3)

Agriculture, livestock (4)

Policy (5)

Entomology, vector-borne diseases (6)

Social and behavioral sciences (7)

Zoonoses, emerging infectious diseases (8)

Geography, Geographic Information Systems (GIS) (9)

Economics (10)

Toxicology (11)

Conservation, wildlife (12)

Plant health (13)

Antimicrobial resistance (AMR) (14)

Law (15)

Public health (19)

Preventive medicine (20)

Molecular biology, genetics & synthetic biology (21)

Urban planning, resources management & disaster management (22)

Qualitative research (23)

Professional skills (16)

No specific emphasis (18)

Other (17)

14 In your current program, is there a practical training experience, internship, or externship required as part of your degree program?

Yes / No

15 Even if not required by your degree program, have you or will you participate in a practical training experience, internship, or externship?

Have participated already prior to program (1)

Required by program and will in the future (2)

Not required, but have already incorporated into program by independent design (3)

Not required, but planning to participate in the future (4)

Have not, and not planning to participate in the future (5)

16 If you have already participated in a practical training experience during the course of your degree program, did you find it to be helpful? Please explain why.

Did not participate, or have not yet participated. (5)

Participated. Yes, it was helpful because...

Participated. No, it was not helpful because...

17 In what type of organization do you hope to find and/or secure a position in the future? Select all that apply.

Academic institution (1)

Local or state government (2)

National or federal government (3)

International organization (4)

Non-governmental organization (NGO) / Non-profit organization (NPO) (5)

Military (9)

Private sector (6)

I plan to be self-employed (7)

If desired, specify a subcategory of one or more categories above (e.g. Ministry of Health) (10)

Other (8)

18 In the future, do you hope to work on challenges that affect countries other than your country of origin or schooling?

Yes / Maybe / No

19 What further education do you plan to pursue in the future, excluding current course of study? Please include any education whether or not it will be related to One Health.

No plans to pursue further education (1)

Undecided (2)

Additional Bachelor's degree (4 years or less) (3)

Master's degree (include MPH, MPVM, etc.) (5)

Professional degree in medicine, veterinary medicine, dentistry, or pharmacology (6)

Other professional degrees (4)

Research based doctoral degree [Doctor of Philosophy (PhD)] (8)

Certificate program (7)

Other (9)

20 What could be better or more comprehensively addressed in your program to help you be better prepared in a career that requires interdisciplinary collaboration among human, animal, plant, and/or environmental health?

21 In the past, did you study in a post-secondary academic degree program with a focus on interdisciplinary collaboration among human, animal, plant and/or environmental health (i.e., One Health)?

Definition of post-secondary: formal education granted after completion of high school, including university and college.

Definition of degree program: grants a formal academic degree after completion, excluding certificates.

Yes / No

22 Why did you decide to pursue your current course of study in One Health? Select all that apply.

Develop professional skills or perform better in job (2)

Change or advance career (1)

Make a more significant contribution to health (4)

Pursue research interest (5)

Increase possibility for better salary and benefits (7)

Recommended by colleagues (6)

Recruited by faculty (8)

Other (9)

23 Was there a specific emphasis or strength in your program?

Epidemiology (1)

Environmental health, ecology (2)

Food safety, food security (3)

Agriculture, livestock (4)

Policy (5)

Entomology, vector-borne diseases (6)

Social and behavioral sciences (7)

Zoonoses, emerging infectious diseases (8)

Geography, Geographic Information Systems (GIS) (9)

Economics (10)

Toxicology (11)

Conservation, wildlife (12)

Plant health (13)

Antimicrobial resistance (AMR) (14)

Law (15)

Public health (19)

Preventive medicine (20)

Molecular biology, genetics & synthetic biology (21)

Urban planning, resources management & disaster management (22)

Qualitative research (23)

Professional skills (16)

No specific emphasis (18)

Other (17)

24 Did you participate in a practical training experience, internship, or externship during your degree program?

Yes / No

25 Was a practical training experience, internship, or externship required as part of your degree program?

Yes / No

26 If you participated in a practical training experience during the course of your One Health degree program, did you find it to be helpful?

Please explain why.

Did not participate (5)

Participated. Yes, it was helpful because... (1)

Participated. No, it was not helpful because... (2)

27 In the past 12 months, what knowledge or skills from your education in One Health do you most often use in the workplace?

Select up to 5 options.

Disease dynamics (4)

Etiology, evolution, and ecology of infectious disease agents (5)

Development of biosurveillance, diagnostics, and/or therapeutic countermeasures (6)

Scientific principles that influence complex challenges in health (e.g. biological complexity, genetic diversity, interactions of systems, etc.) (7)

Cultural and socioeconomic determinants and impacts of illness (1)

Structure and responsibilities of the public health system (8)

Effective identification of, and relationship among local and global key stakeholders in One Health (9)

Interpersonal communication and communication with scientific or non-scientific audiences (10)

Ability to build, work in, and manage a transdisciplinary team, including addressing conflicts (11)

Project management (15)

Cultural awareness (16)

Ability to conduct ethical, scientifically sound research that will inform policy (12)

Ability to conduct qualitative research to study social and behavioral factors (13)

Other (14) ________________________________________________

28 What knowledge and skills from your One Health education have been most valuable? Select up to 5 options.

Disease dynamics (4)

Etiology, evolution, and ecology of infectious disease agents (5)

Development of biosurveillance, diagnostics, and/or therapeutic countermeasures (6)

Scientific principles that influence complex challenges in health (e.g. biological complexity, genetic diversity, interactions of systems, etc.) (7)

Cultural and socioeconomic determinants and impacts of illness (1)

Structure and responsibilities of the public health system (8)

Effective identification of, and relationship among local and global key stakeholders in One Health (9)

Interpersonal communication and communication with scientific or non-scientific audiences (10)

Ability to build, work in, and manage a transdisciplinary team, including addressing conflicts (11)

Program Management (15)

Cultural awareness (16)

Ability to conduct ethical, scientifically sound research that will inform policy (12)

Ability to conduct qualitative research to study social and behavioral factors (13)

Other (14) ________________________________________________

29 In what way did your academic training in One Health benefit you?

________________________________________________________________

30 What skills do you wish had been stronger in your One Health educational experiences, and why?

________________________________________________________________

Start of Block: Section 4 eligibility question

31 Do you currently work in a position that requires interdisciplinary collaboration among human health, animal health, plant health, and/or environmental health (i.e., One Health)? Please include part-time jobs, paid or unpaid internships, externships, and volunteer work. If yes, you may further specify your current area of focus (e.g. climate, nutrition, zoonoses, etc.)

Yes / No

32 In which country do you currently work? If you work in more than one country, choose one primary country where you are physically based to carry out your work for the majority of your time.

▼ Afghanistan (1) ... Zimbabwe (195)

33 In what type of organization do you work? Select all that apply.

Academic institution (1)

Local or state government (2)

National or federal government (3)

International organization (4)

Non-governmental organization (NGO) / Non-profit organization (NPO) (5)

Military (9)

Private sector (6)

I am self-employed (7)

If desired, specify a subcategory of one or more categories above (e.g. Ministry of Health) (10) ________________________________________________

Other (8) ________________________________________________

34 Did you know that there are academic degree programs offered at the undergraduate, Master's or doctoral level with a focus on One Health?

Yes / No

35 Did you receive training related to One Health at the undergraduate or graduate level? For example, taken a course covering topics related to One Health, or attended a program with a One Health approach.

Yes / No

36 If yes, what did you find most useful for your current position in your One Health training? Please select the top 3 options.

I chose "No" in previous question (24)

Epidemiology (1)

Environmental health, ecology (2)

Food safety, food security (3)

Agriculture, livestock (4)

Policy (5)

Entomology, vector-borne diseases (6)

Social and behavioral sciences (7)

Zoonoses, emerging infectious diseases (8)

Geography, Geographic Information Systems (GIS) (9)

Economics (10)

Toxicology (11)

Conservation, wildlife (12)

Plant health (13)

Antimicrobial resistance (AMR) (14)

Law (15)

Public health (19)

Preventive medicine (20)

Molecular biology, genetics & synthetic biology (21)

Urban planning, resources management & disaster management (22)

Qualitative research (23)

Professional skills (16)

No specific emphasis (18)

Other (17) ________________________________________________

37 What are the most important specific knowledge or skills you currently use to carry out your work in One Health?

Select up to 5 options.

Disease dynamics (4)

Etiology, evolution, and ecology of infectious disease agents (5)

Development of biosurveillance, diagnostics, and/or therapeutic countermeasures (6)

Scientific principles that influence complex challenges in health (e.g. biological complexity, genetic diversity, interactions of systems, etc.) (7)

Cultural and socioeconomic determinants and impacts of illness (1)

Structure and responsibilities of the public health system (8)

Effective identification of, and relationship among local and global key stakeholders in One Health (9)

Interpersonal communication and communication with scientific or non-scientific audiences (10)

Ability to build, work in, and manage a transdisciplinary team, including addressing conflict (11)

Program Management (15)

Cultural awareness (16)

Ability to conduct ethical, scientifically sound research that will inform policy (12)

Ability to conduct qualitative research to study social and behavioral factors (13)

Other (14) ________________________________________________

38 What type of training would you have liked to have had that would benefit you in carrying out your current work?

_

39 How did you find your current position?

Select all that apply.

Online public job posting (1)

Mailing list (2)

Internal recruitment within organization (3)

Career fair (4)

Direct contact with employer (5)

Referral from personal contact (6)

Other (7)

40 Did you face any challenges finding your current position?

Yes (please explain) (1) ________________________________________________

No (2)

41 What technical or structural challenges have you had working in the interdisciplinary work among human, animal, plant and/or environmental health in your job setting? Please share some examples.

________________________________________________________________

42 What communication-related challenges have you had working in the interdisciplinary work among human, animal, plant and/or environmental health in your job setting? Please share some examples.

Click to write Choice 1 (1)

Click to write Choice 2 (2)

Click to write Choice 3 (3)

43 What do you think might be some challenges with employing and/or retaining One Health workers, in your workplace or other workplaces?

Start of Block: Section 5 eligibility question

44 Are you currently in a position to employ One Health workers? Definition of One Health worker: An employee whose position requires interdisciplinary collaboration among human health, animal health, plant health and/or environmental health.

o Yes (1)

o No (2)

Start of Block: Section 5: Employers of One Health Workers

45 In what type of organization do you work? Select all that apply.

Academic institution (1)

Local or state government (2)

National/federal government (3)

International organization (4)

Non-governmental organization (NGO) / Non-profit organization (NPO) (5)

Military (9)

Private sector (6)

I am self-employed (7)

If desired, specify a subcategory of one or more categories above (e.g. Ministry of Health) (10) ________________________________________________

Other (8) ________________________________________________

46 How many One Health workers have you hired in the past 24 months?

▼ 0 (21) ... 16+ (39)

47 What are the most important specific knowledge or skills you look for in a One Health worker? Select up to 5 options.

Disease dynamics (4)

Etiology, evolution, and ecology of infectious disease agents (5)

Development of biosurveillance, diagnostics, and/or therapeutic countermeasures (6)

Scientific principles that influence complex challenges in health (e.g. biological complexity, genetic diversity, interactions of systems, etc.) (7)

Cultural and socioeconomic determinants and impacts of illness (1)

Structure and responsibilities of the public health system (8)

Effective identification of, and relationship among local and global key stakeholders in One Health (9)

Interpersonal communication and communication with scientific or non-scientific audiences (10)

Ability to build, work in, and manage a transdisciplinary team, including addressing conflict (11)

Program management (15)

Cultural awareness (16)

Ability to conduct ethical, scientifically sound research that will inform policy (12)

Ability to conduct qualitative research to study social and behavioral factors (13)

Other (14) ________________________________________________

48 What types of training would you like your hires to have?

Select all that apply.

Epidemiology (1)

Environmental health, ecology (2)

Food safety, food security (3)

Agriculture, livestock (4)

Policy (5)

Entomology, vector-borne diseases (6)

Social and behavioral sciences (7)

Zoonoses, emerging infectious diseases (8)

Geography, Geographic Information Systems (GIS) (9)

Economics (10)

Toxicology (11)

Conservation, wildlife (12)

Plant health (13)

Antimicrobial resistance (AMR) (14)

Law (15)

Public health (19)

Preventive medicine (20)

Molecular biology, genetics & synthetic biology (21)

Urban planning, resources management & disaster management (22)

Qualitative research (23)

Professional skills (16)

No specific emphasis (18)

Other (17) ________________________________________________

49 If you have hired one or more One Health workers in the past 24 months, how many of these employees hold an academic degree with a specific focus on One Health?

▼ I have not hired any One Health workers in the past 24 months (1) ... 16+ (18)

50 Do you think that current One Health training programs are producing graduates that meet your needs?

Yes (Which one? Please name academic program, institution & country) (1) ________________________________________________

No (2)

I don't know (3)

51 Do you plan to hire more One Health workers in the future?

Yes (1)

Maybe (2)

No (3)

52 Which platform(s) do you use to recruit One Health workers?

Select all that apply.

Online public job posting (1)

Mailing list (2)

Internal recruitment within organization (3)

Career fair (4)

Direct contact with potential employee (5)

Referral from personal contact (6)

Other (7) ________________________________________________

53 Do you have challenges with recruitment of One Health workers? If yes, please explain what those challenges are.

Yes (1)

No (2)

54 Do you have challenges with retention of One Health workers?

If yes, please explain what those challenges are.

Yes (1) ________________________________________________

No (2)

**End of survey**
